# Combined Tibial Tubercle Fracture With Patellar Tendon Avulsion in an Adult: A Rare Case and Novel Fixation Technique

**DOI:** 10.7759/cureus.7929

**Published:** 2020-05-02

**Authors:** Taylor Woolnough, Gwendolyn Lovsted, Austin MacDonald, Herman Johal, Jamal A Al-Asiri

**Affiliations:** 1 Orthopedics, McMaster University, Hamilton, CAN

**Keywords:** tibial tubercle, patellar ligament, avulsion fracture, tendon rupture

## Abstract

Tibial tubercle avulsion fracture with simultaneous patellar tendon avulsion is a rare injury that has only ever been reported in adolescents; the diagnosis and management of this combined lesion has not been described in the adult population. A healthy 62-year-old male presented with acute knee pain and an inability to walk after a fall on ice. Radiographs demonstrated a displaced fracture of the tibial tubercle; patellar tendon integrity could not be verified by ultrasonography. Intraoperatively, the patient was found to have a distal avulsion of the patellar tendon in addition to tubercle fracture. First, the tendon was secured to the tubercle fragment with transosseous sutures. A novel slotted-plate construct was used to fix the tubercle fragment prior to tightening the sutures. Postoperatively, the patient was permitted to bear weight as tolerated with the operative knee immobilized in extension. After six weeks, knee range of motion was gradually increased using a hinged brace. At one year, the patient had achieved excellent range of motion (full extension to 135 degrees of flexion) and strength (5/5 knee flexion and extension) without residual pain or complications. This case represents the first description of diagnosis, management, and rehabilitation of a combined tibial tubercle fracture with distal patellar tendon avulsion in an adult. The unique construct, a slotted-plate over transosseous sutures, provided excellent results and likely has further applications.

## Introduction

Acute tibial tubercle avulsion fracture is an uncommon injury, typically seen only in adolescents due to the vulnerability of an open physis [1]. In adults, this injury is exceedingly rare, with only several reported cases. Alternatively, the patellar tendon may avulse from an intact tubercle; this injury is similarly infrequent in healthy adults [2,3]. A combined lesion⁠ - tubercle fracture with patellar tendon avulsion from the tubercle fragment - has only ever been reported in adolescents, typically those involved in sport, and can present challenges in both diagnosis and management [4-9]. This is the first reported case of tibial tubercle fracture with simultaneous avulsion of the distal patellar tendon in an adult. The aim of this report is to describe the diagnosis, operative management, and postoperative rehabilitation of a combined tibial tubercle fracture with distal patellar tendon avulsion in an adult.

## Case presentation

**Figure 1:**
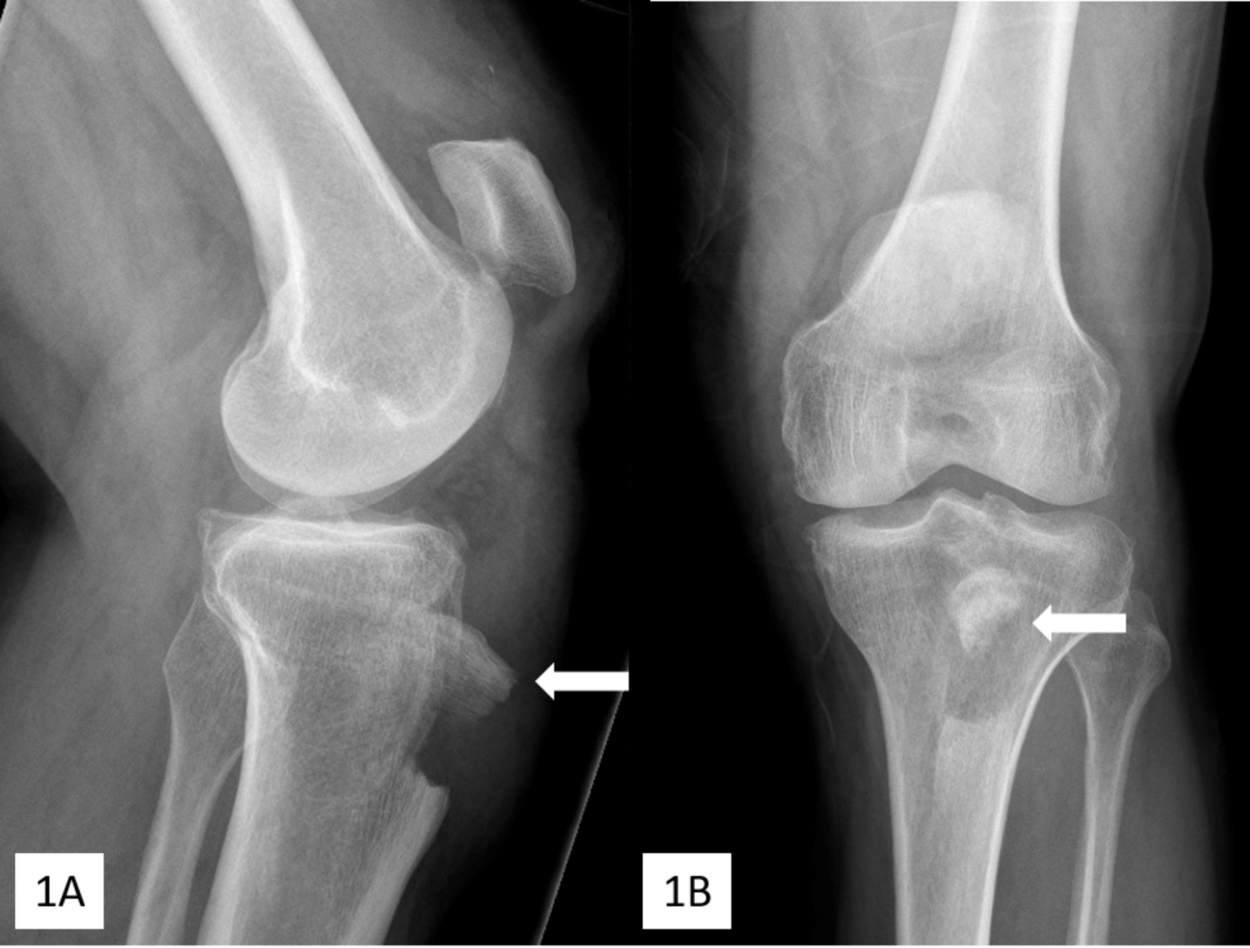
Lateral (A) and anteroposterior (B) preoperative radiographs Avulsion of the tibial tubercle (arrows) and patella alta can be seen; the bony tubercle fragment (arrows) is translated 1.5 cm proximally and is rotated 90º from its anatomic position.

A healthy 62-year-old male hospitality worker presented to the hospital with left knee pain and an inability to walk following a mechanical fall on ice. The patient was healthy and denied previous trauma, and corticosteroid or quinolone use. Examination of the left knee revealed a compromised extensor mechanism and a palpable defect at the inferior insertion of the patellar tendon, with diffuse pain and swelling but no neurovascular or skin compromise. Radiographs demonstrated tibial tubercle avulsion fracture and patella alta (Figure 1). The tubercle fragment was translated 1.5 cm proximally and rotated 90º (Figure 1A).

**Figure 2:**
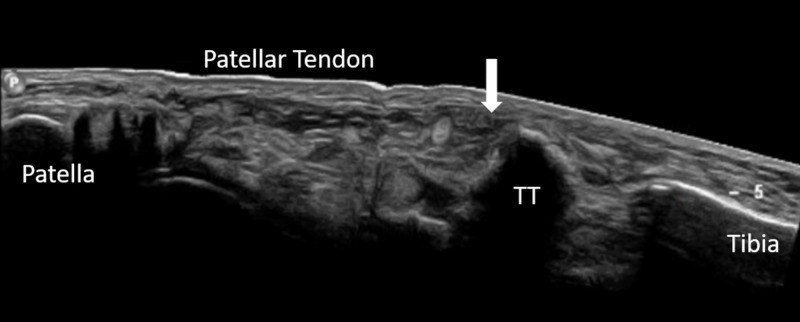
Preoperative ultrasonography Ultrasound demonstrating tibial tubercle (TT) fracture with proximal migration of the patellar tendon and tubercle fragment. Integrity of the distal patellar tendon could not be determined due to hematoma at the site of tendon attachment to the tibial tubercle (arrow).

Ultrasonography demonstrated an intact quadriceps tendon and a normal patellar tendon origin at the inferior patellar pole. The distal aspect of the patellar tendon could not be fully visualized by ultrasound due to overlying hematoma, but proximal retraction was noted (Figure 2). 

**Figure 3:**
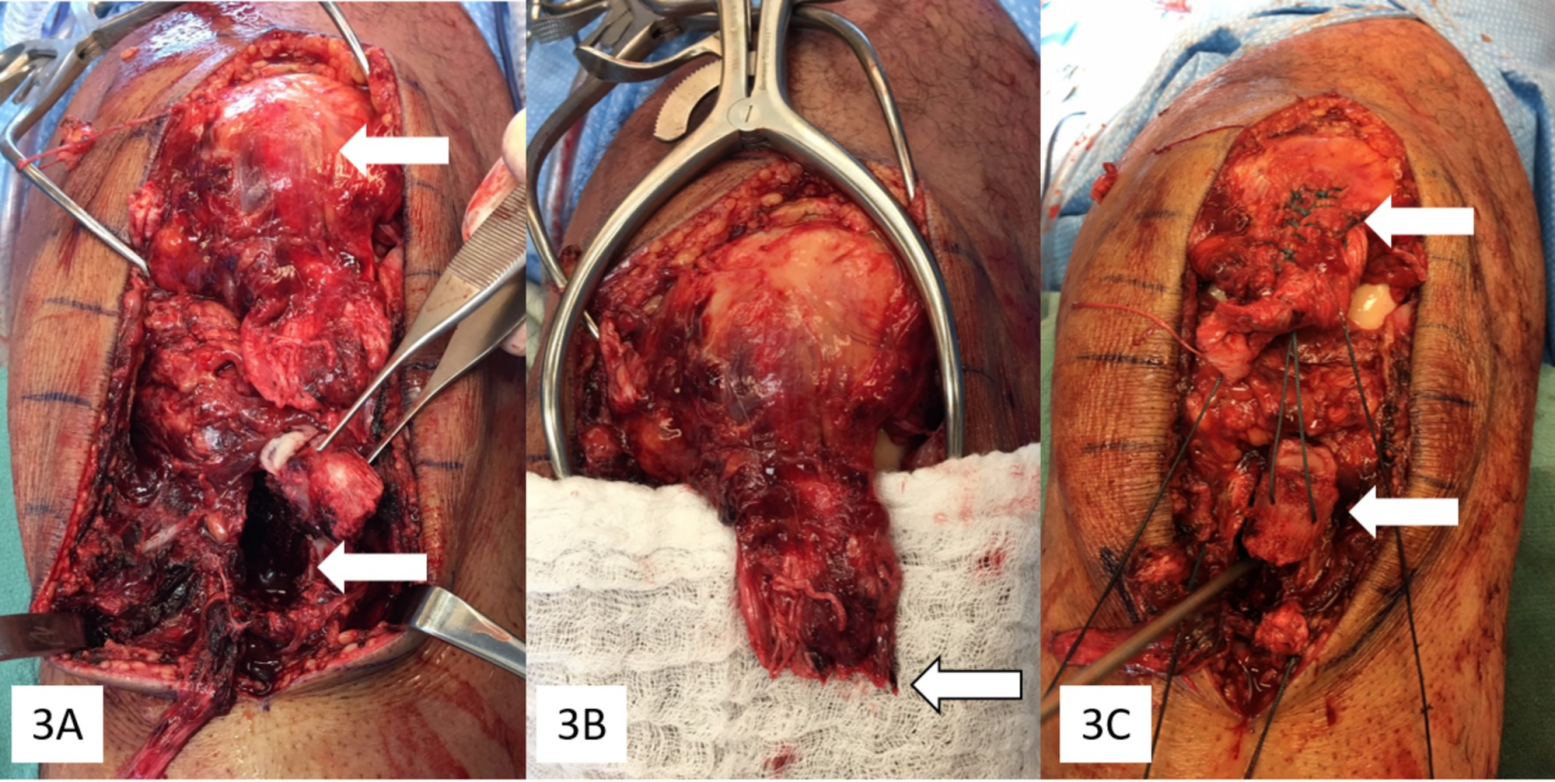
Intraoperative images (A) Free tubercle fragment (in forceps) and tibia donor site (lower arrow) with proximal migration of the patella (upper arrow). (B) Free edge of the avulsed patellar tendon with no remaining bony attachment (arrow). (C) Transosseous sutures through the donor site (metal probe) and tubercle fragment (lower arrow), secured to the patellar tendon with a running-locking technique (upper arrow).

Surgical treatment occurred 24 hours following presentation. An anterior midline approach was used. After excising hemorrhagic prepatellar bursa, evacuating overlying traumatic hematoma, and exposing the fracture donor site (Figure 3A), it was apparent that in addition to the tibial tubercle fracture, the patellar tendon was completely avulsed from the tubercle fragment (Figure 3B). To begin, provisional fixation was achieved with two Kirschner wires. Two transosseous #5 polyester sutures (one medial and one lateral) were placed from intact cortical bone into cancellous bone of the donor site through pilot holes; these sutures were then pushed through the tubercle fragment and were secured through the patellar tendon using a Krackow technique (Figure 3C).

**Figure 4:**
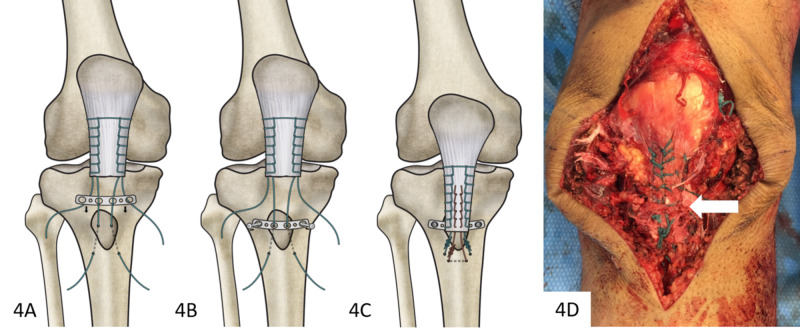
Sequence of plate fixation with final intraoperative appearance (A) A slotted plate was positioned after transosseous suture placement through tibia, tubercle fragment, and patellar tendon. (B) The plate was then secured atop the tubercle fragment. (C) Primary sutures were tensioned and a third, defunctioning suture (red) was placed through a distal pilot hole and secured to the patellar tendon with a running-locking technique. (D) Completed fixation with the tubercle fragment reduced, plate secured (white arrow, not visible), and the tendon reduced.

At this point, slots were cut into the central two holes of a four-hole, 3.5-mm one-third tubular plate. Before tensioning the tendon, this plate was positioned over the reduced tubercle fragment by passing the transosseous sutures through the slots of the plate (Figure 4A). Two 4-mm cancellous screws were used to secure the plate, thereby providing fixation of the tubercle fragment (Figure 4B). The sutures were tensioned and locked, reducing the patellar tendon to the bony fragment and tibia. The primary fixation was reinforced using a third #5 polyester suture, placed through an additional transosseous pilot hole distal to the fracture site and secured to the tendon again using a Krackow technique (Figures 4C, 4D). Medial and lateral retinacular defects were repaired. 

**Figure 5:**
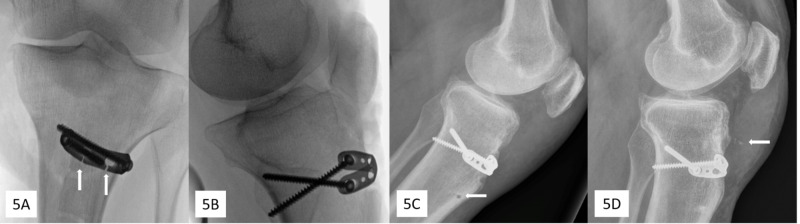
Post-fixation radiographs (A) Intraoperative fluoroscopy demonstrating plate fixation of the tibial tubercle with slots cut in plate (arrows) for passing over sutures. (B) Intraoperative patella alta evident after suture tensioning. (C) Three months postoperative, patella height is appropriate and with visible pilot-hole lucency (arrow) from transosseous suture placement. (D) One year postoperative, slight osseous debris or ossification (arrow) can be seen in the area of the patellar tendon.

Review of final fluoroscopic images confirmed reduction of the tubercle and showed patella baja (Figures 5A, 5B). Two weeks following surgery, the patient began weight bearing in a fixed knee splint. At six weeks, the operative knee could be passively flexed with no pain to 20º so the patient began gradually increasing range of motion in a hinged knee brace, initially locked at full extension. At three months, radiographs demonstrated appropriate patellar height (Figure 5C); the patient had active range of motion from full extension to 120º of flexion with only mild discomfort. At one year, radiographs were unremarkable other than minimal osseous debris and/or ossification (Figure 5D). The patellar height ratio (0.8) was within normal limits using both Caton-Deschamps (normal 0.8-1.2) and Insall-Salvetti (0.75-0.99) methods [10-12]. The patient had full active range of motion (full extension to 135 degrees) and strength (5/5) equivalent to the uninjured knee, with no residual pain, hardware irritation, or bursitis.

## Discussion

This article presents the first case of a combined tibial tubercle avulsion with distal patellar tendon avulsion in an adult. Commonly described mechanisms of tibial tubercle avulsion fracture and patellar tendon avulsion include significant quadriceps contraction with a fixed foot or quadriceps contraction in the presence of an external force acting to produce knee flexion [1-3]. Tubercle avulsion is considerably more common in adolescents than that in adults due to the weakness of an open physis although tubercle fracture may also occur in adults, typically as a result of direct impact [1,4]. Regarding combined lesions, in adolescents it has been proposed that tubercle avulsion is followed by rotation, which tensions soft tissues around the tubercle, arresting fragment translation; continued quadriceps contraction then causes tendon avulsion [5,6]. This mechanism is plausible in our patient as radiographs demonstrated rotation and translation of the bony fragment. While systemic illness (e.g. chronic renal failure, diabetes, chronic steroid use, quinolone treatment, systemic lupus erythematous) may increase risk of tendon rupture, this patient reported no known risk factors [3,4]. 

In patients with radiographic evidence of tibial tubercle fracture, lateral flexion extension radiographs, ultrasonography, and/or magnetic resonance imaging are prudent to rule out concomitant patellar tendon avulsion [5,6]. Previously, isolated distal patellar tendon avulsions have been treated with transosseous sutures or suture anchors [2,3]. In adolescents, combined lesions have been treated using a variety of methods, including staples, wires or suture tape, tension banding, lag screws, suture anchors, and/or transosseous sutures [6,8]. However, the first presentation of this injury in an adult represented a unique challenge as no literature existed describing successful management. Treatment was guided in part by evidence on fixation after tibial tubercle osteotomy; in that context, screw fixation is likely superior to wire fixation [9]. However, screws may have limited purchase in small fragments or osteoporotic bone and may even cause fragment splitting [13]. Plate fixation provided the strength of screw fixation while avoiding potential fragment splitting or loss of screw purchase. In this anatomic location, a one-third semitubular plate was low profile and allowed for easy contouring to the convex surface of the anterior tibia. The slotted plate design allowed precise transosseous suture placement, unobstructed by prior bony fixation. Additionally, placing sutures through the plate provided secure fixation that was not dependent on the adequacy of transosseous tunnels or suture anchors. Fixation with a slotted-plate construct has promising applications outside of the present case, particularly in the setting of tibial tubercle osteotomies and knee arthroplasty where the screws of this construct may be angled to avoid the intramedullary canal, bypassing stemmed or keeled tibial implants.

## Conclusions

This is the first reported case of a tibial tubercle fracture with simultaneous avulsion of the distal patellar tendon in an adult. Despite being the result of a relatively low-energy mechanism, the patient had no known risk factors for tendon rupture. Fixation using transosseous sutures through a slotted plate required no specialized instrumentation and provided an excellent outcome. One year following surgery, the patient had full knee range of motion (full extension to 135 degrees of flexion) and full flexion and extension power (5/5) with no residual pain or other complications. This technique may have additional applications in both trauma and elective procedures.
